# On the Vibration-Damping Properties of the Prestressed Polyurethane Granular Material

**DOI:** 10.3390/polym15051299

**Published:** 2023-03-04

**Authors:** Aleš Gosar, Igor Emri, Jernej Klemenc, Marko Nagode, Simon Oman

**Affiliations:** Faculty of Mechanical Engineering, University of Ljubljana, Askerceva 6, 1000 Ljubljana, Slovenia

**Keywords:** granular material, TPU polymer, vibration damping, energy criterion, lightweight design

## Abstract

Granular materials promise opportunities for the development of high-performance, lightweight vibration-damping elements that provide a high level of safety and comfort. Presented here is an investigation of the vibration-damping properties of prestressed granular material. The material studied is thermoplastic polyurethane (TPU) in Shore 90A and 75A hardness grades. A method for preparing and testing the vibration-damping properties of tubular specimens filled with TPU granules was developed. A new combined energy parameter was introduced to evaluate the damping performance and weight-to-stiffness ratio. Experimental results show that the material in granular form provides up to 400% better vibration-damping performance as compared to the bulk material. Such improvement is possible by combining both the effect of the pressure–frequency superposition principle at the molecular scale and the effect of the physical interactions between the granules (force-chain network) at the macro scale. The two effects complement each other, with the first effect predominating at high prestress and the second at low prestress. Conditions can be further improved by varying the material of the granules and applying a lubricant that facilitates the granules to reorganize and reconfigure the force-chain network (flowability).

## 1. Introduction

Everything around us vibrates; therefore, it is very difficult not to be exposed to vibrations. In general, vibrations can be either structural or acoustic, with the former being the focus of this study. The main sources of structural vibrations are internal combustion engines, compressors, electric motors, fans, and other machinery, but also seismic events. In most cases, vibrations have little impact on human well-being. However, in some cases, such as transportation vehicles, man-operated heavy machinery, or certain manufacturing workplaces, structural vibrations cannot be avoided, but rather they need to be attenuated to provide a certain level of comfort and to comply with work regulations.

The effects of vibrations on the biodynamic response of humans are not yet fully understood; thus, many research efforts are being invested in this important and emerging interdisciplinary field of study. For example, Lindenmann [1] studied how the vibrations of a hammer drill together with the gripping force can contribute to vibration-induced health problems, Liu [2] provided insight into the mechanisms that determine the biodynamic responses of the human body when seated on a rigid seat subjected to vertical vibrations, Zhang [3] proposed a method for designing vibration absorbers for railroad bridges, Sheng [4] proposed a three-directional isolator for seismic vibration suppression, Qin [5] presented current and future challenges with noise and vibration suppression in hybrid electric vehicles, while Du’s study [6] even used deep learning algorithms to improve the vibration comfort of vehicles; and the list goes on.

Recently, economic and environmental arguments have motivated research towards new, lightweight, and high-performance vibration-damping materials, especially in the transportation sector (for comfort and safety), where vibration-damping elements need to be fully integrated into the lightweight support structure or go even further into weight reduction; they should become the support structure. There are not many materials or damping technologies that can fully meet these requirements. A review of the recent literature shows that vibration-isolating materials can be divided into two major groups, namely, metal alloys, including metal matrix composites and thermoplastics, thermosets, and other polymers, including fiber-reinforced plastics and composites. The spectrum of materials and applications used is broad, ranging from cold-formed steel decking for commercial and residential buildings [7], metal matrix composites [8], and lightweight aluminum-based alloys [9] for the aerospace and automotive applications, bitumen, epoxy, butyl rubber, and piezoelectric-based viscoelastic damping materials [10,11] for the automotive, medical, and consumer goods applications to fiber-reinforced polymers [12] and laminated composites [13,14] for the automotive, aerospace, and marine industries. Recently, reports on new lightweight porous materials such as elastic lattice metamaterials [15] and composites with bio-ingredients [16] have been published, essentially confirming the topicality of the research. However, when both vibration damping and supporting functions are required, most materials cannot provide the expected performance.

Nevertheless, as shown by this research, the expected performance can be achieved by using the novel granular-based vibration-damping technology. This research is important because it shows that the technology can both improve vibration-damping performance and load-carrying capacity at low levels of prestress and reduce the overall mass of the damping elements. The details of the research are presented in the continuation of the paper.

## 2. Materials and Methods

### 2.1. Research Thesis and Motivation

Vibration damping performance is an essential mechanical property inherent in any material. Materials with low damping capacity absorb less energy (corresponding to a short delay between excitation and response) than materials with high damping capacity, which can absorb more energy before releasing it back into the environment, for example, in the form of heat [17]. Polymers (i.e., composites with additives and fillers or certain laminates), with their long-chain molecular structure, are typical viscoelastic materials with a high energy dissipation factor and are, therefore, very convenient for practical vibration-damping applications. However, since load-carrying capacity and vibration-damping properties are often mutually exclusive [18], a balance must always be found between strength, durability, weight-to-load ratio, as well as the increasingly important aspects of price and environmental impact [19], and damping performance [20]. This paper presents the results of an experimental investigation of the vibration-damping properties of prestressed polymeric thermoplastic polyurethane (TPU) in granular form, which can be used in many potential applications in lightweight and/or environmentally sustainable applications, e.g., for a new generation of vibration and shock absorbers with properties that exceed the limits of many current solutions.

Our research follows patented inventive ideas proposed by Emri et al. [21,22,23,24,25,26], referred to as the “high-pressure force-network technology” that utilizes two energy dissipation mechanisms acting at two different time-space scales, as follows:

(i) The molecular-scale—interactions between molecules, governed by the pressure–frequency (time) superposition principle [21,22,23,24], and
(ii) The macro-scale—interactions between granules in a granular system, forming a force-chain network [25,26].

The two mechanisms complement each other. The macro-scale force-chain network mechanism works well at low hydrostatic pressure, while the molecular scale mechanism prevails at high hydrostatic pressure. The solution, according to the invention, is to use granular materials for which it is true that the shear stresses within the material decrease faster than the principal stresses as the particles become smaller. At the same time, analysis of flowability has shown that granular materials, when excited beyond a certain stress level, flow similarly to liquids while retaining all the properties of bulk material. With this innovative idea, it is possible to improve damping properties by up to 100 times and stiffness by up to 10 times when subjected to a certain high level of prestress. This is because prestressed granules with the right particle size distribution can exert hydrostatic pressure on themselves, which in turn affects the damping properties and stiffness [27].

The aim of this research is to confirm experimentally that the two mentioned mechanisms complement each other and that the invented technology is suitable to dampen vibrations and/or shock loads also at lower levels of prestress.

Finally, the expected outcomes of the research could also inspire the development of a new generation of high-performance, lightweight granular-based vibration dampers for passenger safety and comfort, impact, seismic load-damping, and many more.

The rest of the paper is organized as follows. Section 2.2 presents the theoretical background of the two granular-based vibration-damping technologies. In Section 2.3, the experimental setup and material selection are described in detail. The results are presented in Section 3. The last section is devoted to discussion.

### 2.2. Theoretical Background

#### 2.2.1. Particle Damping Technology and Dissipative Bulk and Granular Systems Technology (DBGS)

The particle damping technology, as used in particle dampers (i.e., containers partially filled with granules attached to the vibrating structure) for use in lightweight structures to improve their dynamic behavior and not contribute significantly to their weight, is not new [28]. The mechanism of this technology is essentially based on energy dissipation through inelastic collisions and friction between the granules and in interaction with the walls of the container [29], as well as momentum exchange and acoustic radiation [30]. In order to keep the mass low, the containers can also be made of lightweight structures, e.g., honeycomb sandwich panels [31]. From a recent systematic review of particle damping modeling and testing by Gagnon [32], it appears that particle dampers are very popular due to their advantages, such as resistance to harsh conditions, damping of a wide frequency spectrum, insensitivity to ambient temperature, high reliability, and low cost, to name a few. This type of (passive) vibration damper is valued in many industries, such as automotive, aerospace, and power, with a wide range of applications. However, due to the nonlinear nature of the damping behavior [33], both the analysis and design of such dampers are challenging, i.e., a great experimental effort is required to derive equivalent models and/or additional extensive computations are required when detailed physical models with a large number of contact equations are used to predict the damping performance, as concisely summarized by Oltmann [28]. Particle dampers also have other shortcomings; for example, unused material in lightweight construction generally means indirect energy loss, which in turn limits their applicability. In most cases, the particle damper is attached to the main structure, such as an internal combustion engine [28], aircraft components [34], or printed circuit boards [35], without any real load-carrying capability, so the benefits of having such a device on board should be carefully weighed. In addition, damping performance is limited by the nonlinear behavior of the damper, and there are many influencing parameters that affect performance that cannot be easily accounted for [29]. Gravity can also affect the damping performance by demobilizing the granules [36], so particle dampers may perform better under microgravity conditions and in space applications. For this reason, particle dampers are also less suitable when relatively small vibrations need to be attenuated (low momentum transfer, lower dry friction, and lower shock losses), such as in the protection of civil structures, as shown by Lu [37].

In contrast to the particle damping technology described above, the Dissipative bulk and granular systems (DBGS) technology could provide both vibration-damping capability and a viable option for practical implementation of the damping element as a load-carrying component of the lightweight structure. Both can be justified as follows.

Polymeric and elastomeric materials with good damping properties generally have low stiffness, which is often insufficient to support the load. In addition, with such materials, the highest vibration damping is often achieved at excitation frequencies that are outside of practical requirements. Therefore, to improve stiffness and retain as many damping properties as possible, elastomeric materials are combined with other materials, such as metal or fiber–resin layers [38]. From a technical, economic, and environmental point of view, it is better to work with “pure” material than with composites. For example, Emri [25] proposed that both stiffness and damping properties of certain polymers can be improved at the same time by increasing the level of prestress to the material aiming to establish hydrostatic conditions. The research-based invention demonstrated that hydrostatic conditions can alter damping properties, particularly in terms of improving damping performance for several orders of magnitude at lower frequencies. Highly prestressed granular materials exhibit some interesting phenomena, namely flow-like properties that bulk materials do not have while retaining all other properties of bulk materials. This motivated the development of an apparatus to measure friction in granular materials [39], followed by fundamental research on the vibration-damping properties of hydrostatically prestressed TPU material [27] and led to the recent study by Venkatesh [40] on the effects of particle size distribution on the flowability of uniaxially compressed granular materials, which is important for understanding the formation of force-chain networks [21,22,23,24].

In continuation of this paper, we provide a brief overview of the theoretical background of the effect of pressure on the behavior of time-dependent materials and on the flowability of granular materials. A more comprehensive review can be found elsewhere [39,40,41,42].

#### 2.2.2. Effect of Pressure

**Figure 1:**
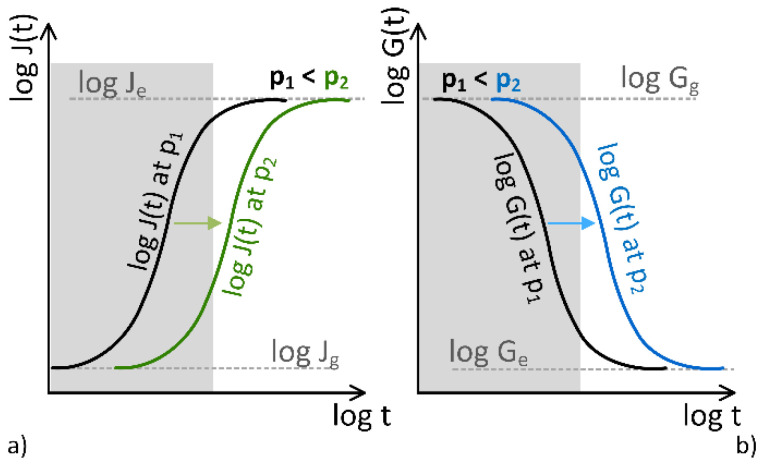
Schematics present behavior of cross-linked elastomers under pressure with a shift of the (**a**) creep compliance and (**b**) the relaxation modulus towards longer times.

Since hydrostatic pressure is fundamentally a 3D stress, i.e., $p=\sigma_{kk}$, it can be expressed essentially as a stress load. Polymers are affected by pressure in both solid and molten states. The local motion of polymer molecules is restricted, and even relative motions with respect to each other are prevented when the material is simultaneously pressurized (state with pressure load) and subjected to an external strain or stress. As a result, the rearrangement of the molecules triggered by the external load is slowed down. On the macro level, this can be observed as a slowing of the creep and relaxation processes. Thus, changes in the macroscopic time dependence of polymers are caused by hydrostatic pressure. Figure 1 shows graphically how pressure affects the creep (a) and relaxation (b) process. As can be seen from the figure, higher pressure, $p_{2}>p_{1}$, leads to a shift in material characteristics towards longer times, demonstrating that all molecular processes are equally affected by the applied pressure. In addition, an increase in pressure does not change the shape of the response functions but only shifts them in parallel along the logarithmic time-axis. Assuming that the gray areas in Figure 1 indicate the lifetime of a material and assuming that an increase in pressure shifts the response functions (creep and relaxation) to longer times, it may be concluded that exposing material to sufficiently high (hydrostatic) pressure the properties related to its glassy state may prolong the expected lifetime of a product.

On the macro scale, the increase of pressure affects the mechanical properties of the material in the same way as the decrease in temperature, namely by shifting the response functions horizontally along the logarithmic time scale. Therefore, the time–temperature superposition [43] may as well be extended to pressure (principle of time–pressure superposition). However, it needs to be noted that on the molecular scale, time and pressure processes are not the same. Temperature governs changes in the average kinetic energy of the polymeric molecules, while pressure does not change the energy state of the molecules, but it locally restricts the kinematics of molecules. Using the time–pressure superposition, segments of principle response functions, measured at different isobaric conditions, can be stacked into a single “master curve”. Such a curve spans several time decades, which is longer than what can normally be measured in real-time experiments. It must be pointed out that the time–pressure superposition principle can only be applied to piezo-rheological simple materials, i.e., materials in which all molecular groups respond equally to the applied external load (pressure). Time–pressure superposition has not (yet) been sufficiently researched, so it is not clear whether it also applies to multi-phase materials, such as block/graft copolymers, hybrid materials or polymeric blends, and bituminous materials [43].

##### Modeling the Effect of Pressure

Several proposed models are able to account for the effect of pressure on the mechanical properties of polymers. The most universal of these models are FMT model proposed by Fillers, Moonan, and Tschoegl [44] and the Knauss–Emri (KE) model [45], both of which consider the simultaneous effect of temperature and pressure. The FMT model is used to describe the effects of pressure and temperature at equilibrium, while the KE model can be used for modeling material behavior under varying temperatures and pressure, i.e., for conditions where the material is not in thermodynamic equilibrium. It is important to note that at constant pressure and temperature, the KE model reduces to the equations of the Williams, Landel, and Ferry (WLF) and FMT models.

A crash or impact loading is a time-varying loading situation; the appropriate model to use is the KE model [45], where stress–strain relationships are expressed by a deviatoric part, $S_{ij}\left(t\right)$ related to changes in shape, and a dilatometric part, $\sigma_{kk}\left(t\right)$ related to the changes in volume, as given by the following equations:

(1) $$\sigma_{kk}\left(t\right)=3\int_{0}^{t}K\left[t^{\prime }\left(t\right)-\xi^{\prime }\left(\xi \right)\right]\frac{\partial \theta \left(\xi \right)}{\partial \xi }d\xi ,$$

(2) $$S_{ij}\left(t\right)=2\int_{0}^{t}G\left[t^{\prime }\left(t\right)-\xi^{\prime }\left(\xi \right)\right]\frac{\partial e_{ij}\left(\xi \right)}{\partial \xi }d\xi ,$$

where

(3) $$t^{\prime }\left(t\right)-\xi^{\prime }\left(\xi \right)=\int_{\xi }^{t}\frac{du}{\phi \left[T\left(u\right),\theta \left(u\right)\right]},$$

(4) $$\log\phi \left[T\left(u\right),\theta \left(u\right)\right]=\frac{b}{2.303}\left[\frac{1}{f\left[T\left(u\right),\theta \left(u\right)\right]}-\frac{1}{f_{0}}\right],\;\mathrm{and}$$

(5) $$f\left[T\left(u\right),\theta \left(u\right)\right]=f_{0}+\int_{0}^{u}\alpha \left(u-\lambda \right)\frac{\partial T\left(\lambda \right)}{\partial \lambda }d\lambda +\frac{1}{3}\int_{0}^{u}B\left(u-\lambda \right)\frac{\partial \sigma_{kk}\left(\lambda \right)}{\partial \lambda }d\lambda .$$

Here, K(t) and G(t) denote the time-dependent bulk and shear moduli, and $B\left(t\right)=K^{-1}\left(t\right)$ denotes the time-dependent bulk creep compliance (an inverse of the time-dependent bulk modulus), $f_{0}$ and $\alpha_{f}\left(t\right)$ denote the fractional free volume and the corresponding time-dependent volumetric thermal expansion coefficient (determined at a reference temperature $T_{0}$ and pressure $p_{0}$). A dedicated experimental setup, developed by Moonan and Tschoegl [44] and later upgraded by Kralj, Prodan, and Emri [46,47], is used to measure the material functions of the KE model.

#### 2.2.3. Flowability of Granular Systems

We have found [21,22,23,24,25,26] that granular viscoelastic materials with a selected multimodal size distribution exhibit fluid-like behavior while retaining the properties of the bulk material from which they are made. Therefore, they can be considered a “pressure medium” that exerts hydrostatic pressure on itself within a container, thereby modifying its intrinsic damping properties. In order to establish hydrostatic pressure in the granular system, one should be able to measure the flowability (readiness to flow) of granular material at rest, known as zero-rate fluidity. An instrument, the Granular Friction Analyzer (GFA), has been developed to measure the fluidity of granular systems at rest [39,40,42].

**Figure 2:**
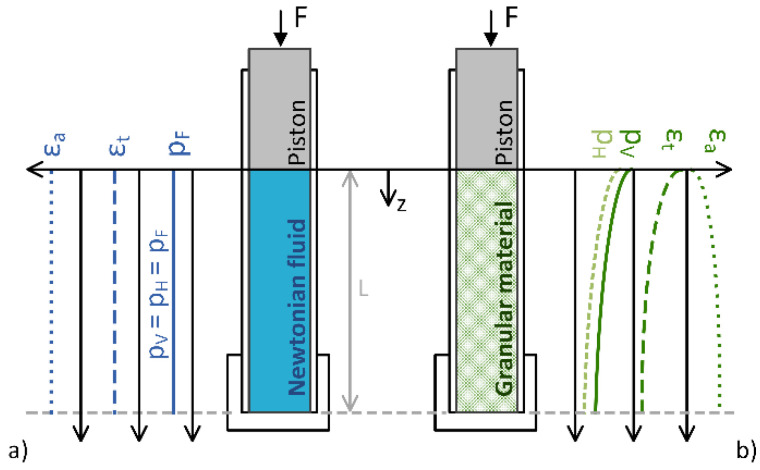
Graphical presentation of vertical $p_{V}\left(z\right)$ and horizontal $p_{H}\left(z\right)$ distribution of internal pressure, the distribution of axial $\varepsilon_{a}\left(z\right)$ and tangential $\varepsilon_{t}\left(z\right)$ strains at uniaxial compression in the case of (**a**) Newtonian fluid and (**b**) granular material.

The design of the Granular Friction Analyzer apparatus follows the realization that Newtonian fluids have the unique property of distributing the forces acting on them uniformly in all directions throughout the occupied volume. Thus, if we subject a Newtonian fluid in a closed cylinder to a uniaxial pressure load F applied by a piston, it can be observed that the distribution of pressure is constant in all directions, $p_{F}=F/A=p_{V}=p_{H}$, as graphically shown in Figure 2a. On the other hand, if granular material is subjected to the same load, the distribution of pressure in the axial *z*-direction decreases with distance from the piston (Figure 2b). The distribution of pressure cannot be measured directly. Instead, it can be measured indirectly, for example, by measuring the corresponding deformation of the cylinder surface in the axial, $\varepsilon_{a}\left(z\right)$, and tangential, $\varepsilon_{t}\left(z\right)$, directions along the axis of the cylinder by using strain gauges [39,42] or the digital image correlation (DIC) technique [40]. A GFA index has been introduced as a measure of the flowability of granular materials, which allows characterizing the flowable behavior of granular materials under uniaxial compressive loading. The fundamental part of the GFA index calculation represents the integration of the internal pressure distribution along the cylinder wall in which the granular material is uniaxially compressed by a piston. The value is further normalized by the pressure distribution of a Newtonian fluid [39,40,42].

#### 2.2.4. Utilizing Hydrostatic Pressurization

**Figure 3:**
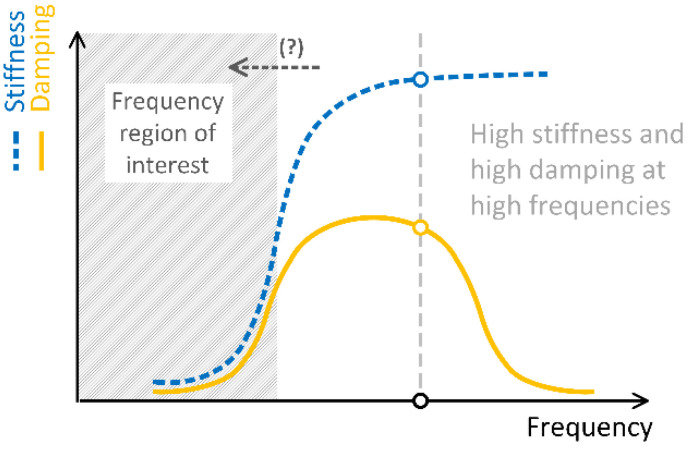
Frequency with damping performance of materials for vibration and impact isolation.

Existing structural and vibration damping solutions cannot fully exploit the damping properties of time and frequency-dependent materials since their maximum damping properties are at frequencies far from the frequency range of interest to engineers, as schematically depicted in Figure 3.

The frequency domain functions may be obtained by the interconversion from the corresponding creep and relaxation functions measured in time domain [48],

(6) $$G^{\prime }\left(\omega \right)=\omega \int_{0}^{\infty }G\left(t\right)\sin\omega t\;dt,\;G^{\prime \prime }\left(\omega \right)=\omega \int_{0}^{\infty }G\left(t\right)\cos\omega t\;dt$$

and

(7) $$J^{\prime }\left(\omega \right)=\omega \int_{0}^{\infty }J\left(t\right)\sin\omega tdt,\;J^{\prime \prime }\left(\omega \right)=\omega \int_{0}^{\infty }J\left(t\right)\cos\omega t\;dt$$

where $G^{\prime }\left(\omega \right)$ and $J^{\prime }\left(\omega \right)$ denote the storage modulus and storage compliance, respectively, and $G^{\prime \prime }\left(\omega \right)$ and $J^{\prime \prime }\left(\omega \right)$ denote the loss modulus and the loss compliance, respectively. The first two represent the stiffness of the material, and the last two represent its damping and energy absorption capabilities.

The properties of materials subjected to hydrostatic pressure have been shown to shift along the time and frequency (logarithmic) scales. In the time domain, an increased pressure shifts material properties toward longer times, while in the frequency domain, all four material functions, namely storage modulus and storage compliance, and loss modulus and loss compliance, are shifted to the left, in the direction of lower frequencies, as shown by the arrow in Figure 3. Thus, by properly selecting the hydrostatic pressure to which the material is subjected, the frequency range of its maximum damping properties can be matched to the resonant frequency of the structure or to the velocity of an impact load. In this way, the damping properties of the damping material can be fully utilized, and the energy absorption properties of a vibration damper can be maximized.

However, how can hydrostatic pressure be generated in a vibration-damping material? The simplest technical solution would be a uniaxial or biaxial load, but this is not possible. Since pressure is a sum of principal stresses, $p=\sigma_{11}+\sigma_{22}+\sigma_{33}$, as soon as the load is not spatial (three-dimensional), shear stresses are generated. If the shear stresses reach a critical value before the pressure required to shift the material functions is reached, the damping material fails.

As mentioned in Section 2, the solution according to the invention finds application in granular materials, for which it is true that the smaller the particles are, the faster the shear stresses in the material decrease compared to the principal stresses.

(8) $$\underset{V\to 0}{\lim}\frac{\sigma_{kk}}{S_{ij}}=\infty .$$

At the same time, analysis of flowability has shown that granular materials, when loaded beyond a threshold level of stress, flow similarly to liquids whilst retaining all the properties of bulk material. Therefore, multimodal elastomeric granules from micro to macro size can be employed as a pressure medium (similar to air in tires) to exert hydrostatic pressure on themselves and modify the frequency dependence of their energy absorption properties. Even the stiffness of the damping element (again, similar to air in tires) can be adjusted by a suitable pressure setting. Such damping elements, as shown in a patent [21,22,23,24,25,26], consist of elastomeric granules enclosed in a flexible sleeve made of rigid fibers [41].

**Figure 4:**
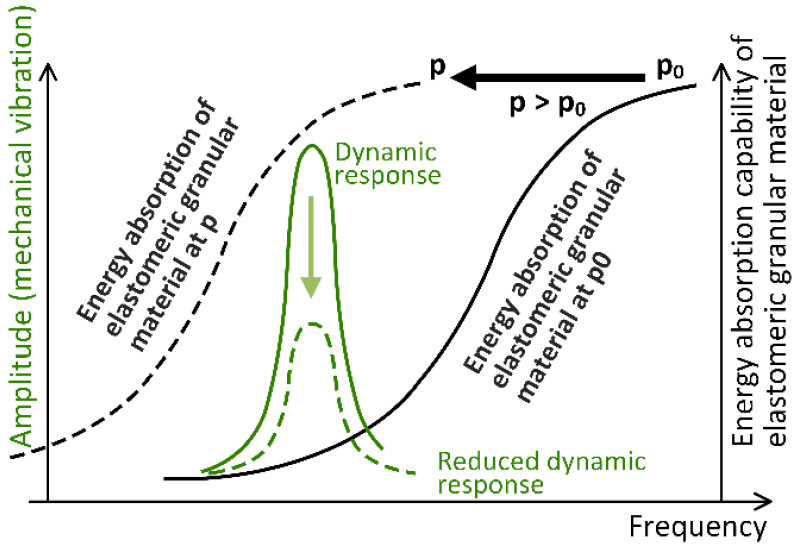
Schematic presentation of the damping element working principle.

The operating principle of the patented damping element is shown graphically in Figure 4. The bell-shaped green solid line shows the dynamic response of a structure, and the dashed green line represents the decreased dynamic response of the structure after applying the patented damping elements. The solid black line on the right side of Figure 4 shows the frequency dependence of energy absorption of an elastomeric granular material at atmospheric pressure $p_{0}$. If the same elastomeric material is subjected to a suitable pressure $p>p_{0},$ its damping properties can be adapted to match the excitation frequency (black dashed line in Figure 4), which in turn effectively reduces the dynamic response of the structure.

### 2.3. Experimental Section

In this section, the employed experimental equipment and procedures, as well as specimen preparation, are presented. Although the research is concerned with determining the vibration-damping properties of the selected granular material, it should be reiterated at this point that the core of the investigation is committed to practical applications, such as the implementation of the DBGS technology in lightweight vibration dampers or shock absorbers. The material selection is described first, followed by presentation of procedure to prepare specimens, presentation of the developed filling tool, and the employed loading conditions.

#### 2.3.1. Material Selection

Material under investigation is TPU Elastollan^®^ (manufactured by Basf^®^) 1190A and 1175AW in Shore hardness grades 90A and 75A, respectively. The TPU material was selected upon previous research of Bek [27], which revealed that this material exhibits sensitivity to pressure, which is an important material property for this research. In addition, the two grades of TPU material were selected because Bek’s research also revealed that 1190A material has higher sensitivity to pressure than 1175AW, which in turn means that the stiffness and energy absorption of the former is several decades better compared to the latter.

The TPU material was supplied already granulated, with the granules having the shape of an elongated spheroid. The typical size of a single granule is approximately 4.95 mm about the long axis and 3 mm about the shorter axis, and it weighs 0.03 g. The material was always stored in its original resealed package bag in a dry room. Other physical properties of the two materials can be found online on the manufacturer’s websites [49], but some relevant values are also presented here in Table 1.

#### 2.3.2. Granular Material Container

Based on previous experience with granular material, two completely different designs of the specimen were considered, namely specimens with metal casing as used by Venkatesh [40] or specimens with braided synthetic fiber sleeves as used by Bek [27]. It was decided that the latter option offers better weight-to-filling ratio and promises more options to test the damping performance of the granular material as compared to the metal specimen type, which is currently the most viable option for other types of experimental investigation, such as measurements of flowability and internal friction at high pressure.

**Figure 5:**
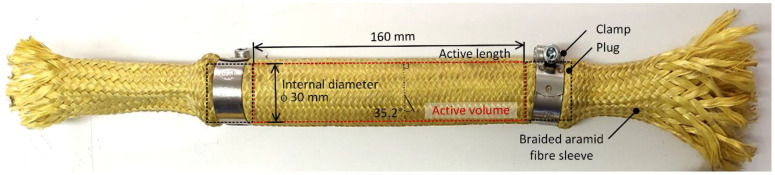
Braided aramid fibers sleeve specimen, including two stainless steel clamps and two plugs.

The employed synthetic textile sleeves are made of aramid (DuPont Kevlar^®^) fibers braided by continuous filament in a cylindrical open ends pattern, as shown in Figure 5, with fibers arranged at an angle of 45° about the sleeve axis. The nominal inner diameter of an empty sleeve is 25.4 mm (1″), and the diameter of the filament is one millimeter. The sleeves were cut to a free length of 300 mm. Due to the braiding pattern, such a sleeve can assume different sizes and cylindrical shapes by changing the ratio between the diameter and the length of the sleeve. Since the internal volume should be kept as constant as possible during the experiment, a limiting angle was calculated at which the sleeve has the maximum volume. A simple comparison between the volumes at different lengths and diameters resulted in the sought angle of 35.2°. At this angle, the inner diameter of the sleeve equals 30 mm. To close the sleeve, we used aluminum plugs with a machined groove for a metal clamp, which ensures a properly tight connection of the sleeve to the two plugs. In our experience, such a connection provides an excellent seal and prevents any axial movement of the plug, even under high prestress or high internal pressure. In addition, the use of plugs allows convenient specimen preparation, as well as controlled administration of initial prestress. A manual hydraulic press was used to apply sufficient force to close the sleeves. The active length, which is the internal distance between the plugs and defines the volume filled with granules, was carefully set at 160 mm for all specimens.

An important aspect of the investigation of material response is the application of an external load. It was assumed that the use of braided sleeves would allow the load to be applied more directly to the granular material, which on the one hand, allows more attention to the response of the granular material and, on the other hand, pays less on the effects of the container, as well as allowing greater flexibility with load cases.

#### 2.3.3. Filling Device

**Figure 6:**
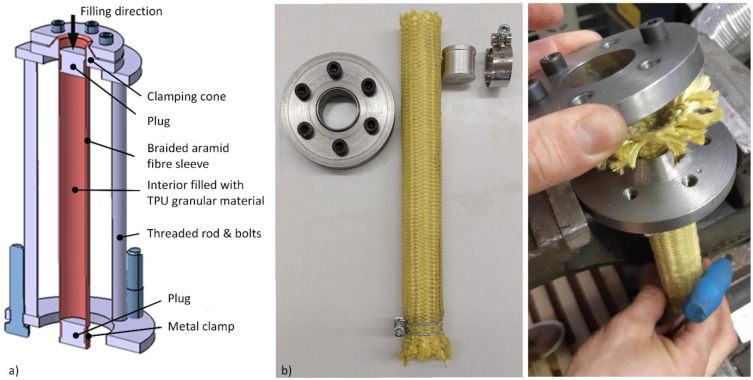
Detailed images of (**a**) the developed metal tool to prepare the granular specimens and (**b**) specimens preparation procedure.

Braided sleeves have no self-supporting stiffness; hence, in order to ensure good reproducibility of specimens, a metal tool was developed to assist in sample preparation. The tool consists of a pair of clamping cones, and an upper and a lower plate connected by threaded rods and bolts. The developed metal tool for preparing the granular specimens is depicted in Figure 6a. The clamping cones are used to hold the sleeve firmly in the position during it is being filled with granules, while the threaded rods are used to allow filling of sleeves of different lengths and to ensure a constant distance between the plates.

Because all specimens were made with the same internal length, the only way to have specimens with different prestress is to vary the amount of the content. By amount, it is meant the mass or volume of granular material that is put into the sleeve. No prestress is achieved when granular material is filled into the sleeve’s active length volume and closed with plugs (see Figure 6b). If prestress is needed, additional volume of material should be added. In order to control how much volume of the sleeve is actually filled by the material and how much of the volume represents vacancies between the granules, a volume ratio κ has been introduced as given by Equation (9). Where $V_{\mathrm{TPU}}$ represents volume of the sample TPU material, defined by the sample mass $m_{\mathrm{TPU}}$ and relative density $\rho_{\mathrm{TPU}}$. $V_{0}$ is a reference volume. If κ equals 100%, the reference volume is completed by the material.

(9) $$\kappa =\frac{V_{\mathrm{TPU}}}{V_{0}}\cdot 100\%=\frac{m_{\mathrm{TPU}}}{\rho_{\mathrm{TPU}}\cdot V_{0}}\cdot 100\%$$

**Figure 7:**
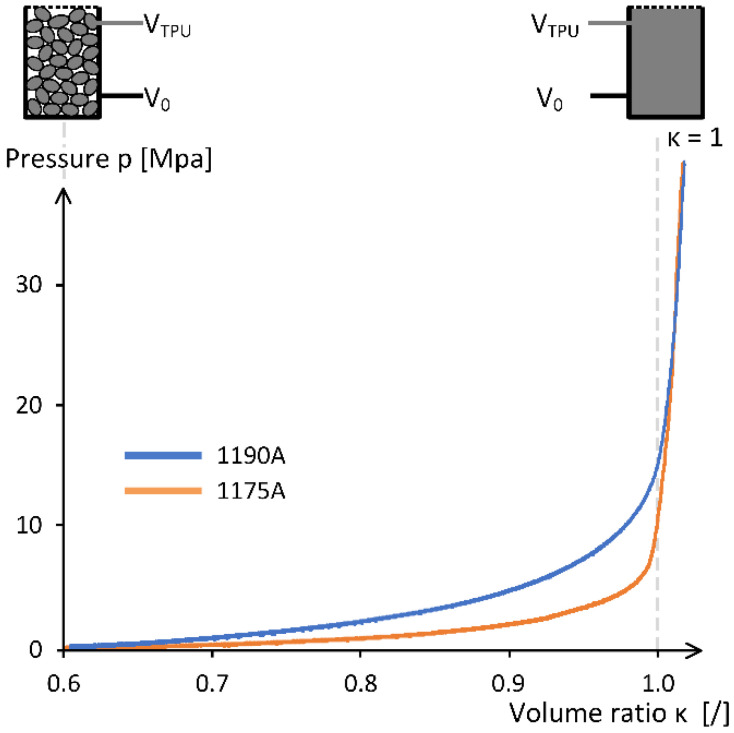
Level of prestress (pressure) with respect to volume ratio κ for both materials.

It should be noted, however, that the volume ratio, as given by Equation (9), tells nothing about the actual arrangement of granules within the volume or the so-called void ratio. The value of the void ratio depends on how the granules naturally occupy the given volume, which is rather stochastic process of particle arrangement that is mainly influenced by the size, shape, and specific gravity of granules [50]. The amount of prestress is thus directly related to the difference between the volume ratio and the void ratio, i.e., a volume ratio of 0.8 at a void ratio of 0.5 means higher prestress than a volume ratio of 0.8 at a void ratio of 0.7. The measured relationship between the level of prestress and the volume ratio is shown in Figure 7. The diagram shows that as the level of prestress increases, the number of voids between the granules decreases, and the volume of the TPU material eventually reaches the volume of the container (*κ* = 100%). After that, the volume of the material starts to change. It should be noted that in the case of no prestress, when the pressure is zero (the material is only filled into the container), the volume ratio is equal to the void ratio.

When preparing specimens without initial prestress, it was observed that the filling process sometimes resulted in inconsistent values of κ. Best repeatability was obtained when the specimens were gradually filled with small amounts of material and then compacted (e.g., with a vibrator). In this case, values of 0.627 and 0.621 were calculated for 1190A and 1175AW TPU material, respectively.

To improve the void ratio, we added a small amount (2.5% by mass) of industrial petroleum jelly (Vaseline) to the material before filling. It was found that the void ratio improved, resulting in 0.643 and 0.667 for 1190A and 1175AW TPU material, respectively. It should be noted that the consistency of the measurements was also improved. Therefore, it was decided to extend the research by including the lubricated specimens. To this end, two other volume ratios, 80% and 93% were chosen for testing specimens with initial prestress. The latter seems strange, but this is as much as the setup as well as the aramid fiber sleeve allowed during the filling process before breaking apart. In addition, it was observed that the granules without Vaseline quickly became immobile when filled into the sleeve and could not flow further, even when the material was added in small amounts gradually. For this reason, only the specimens without prestress were tested with and without Vaseline, while the others were always lubricated. Four specimens were prepared for each material. In order to avoid misunderstanding and to allow a better visual presentation of the results, specimens were classified by category, where each category represents a different set of materials, filling conditions, and initial prestress.

Assuming 160 mm of active length and internal diameter of 30 mm, we can then interpret the volume ratios in terms of mass of used granular material as follows from Equation (9) and as given in Table 2.

#### 2.3.4. Experimental Setup and Testing Conditions

The experimental evaluation of the material and damping properties was carried out in a systematic and step-by-step manner, from the testing of the base material to the testing of the test specimens, as presented in this section. In addition, details of the experimental setup and test conditions are presented.

**Figure 8:**
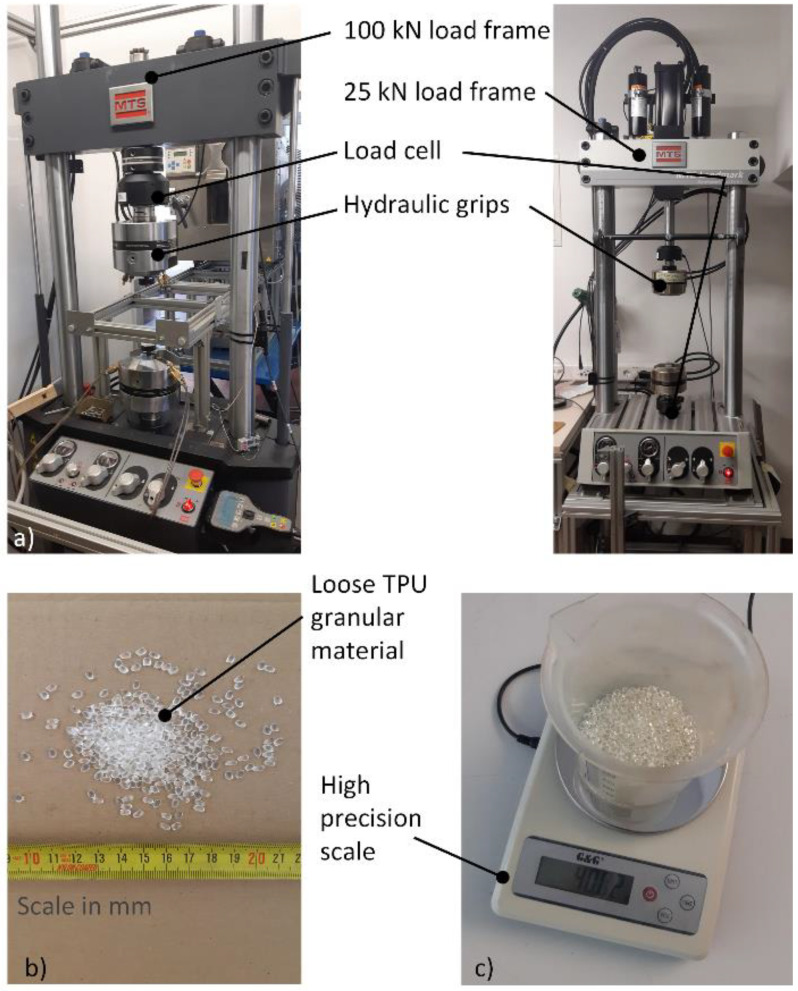
Experimental setup including (**a**) servo-hydraulic load frames, (**b**) TPU granular material and (**c**) a high precision scale.

All experiments were conducted on the MTS Landmark servo-hydraulic system with two load frames, namely a 100 kN and a 25 kN load frame. Experiments with lower loads were performed on the 25 kN load frame with a 5 kN load cell, while high energy experiments were performed on the 100 kN load frame with a 100 kN load cell. A special fixture was used to hold and load the specimens, as shown in Figure 8. A high precision scale (500 g range, 0.01 g division) was used to weigh the granules.

**Figure 9:**
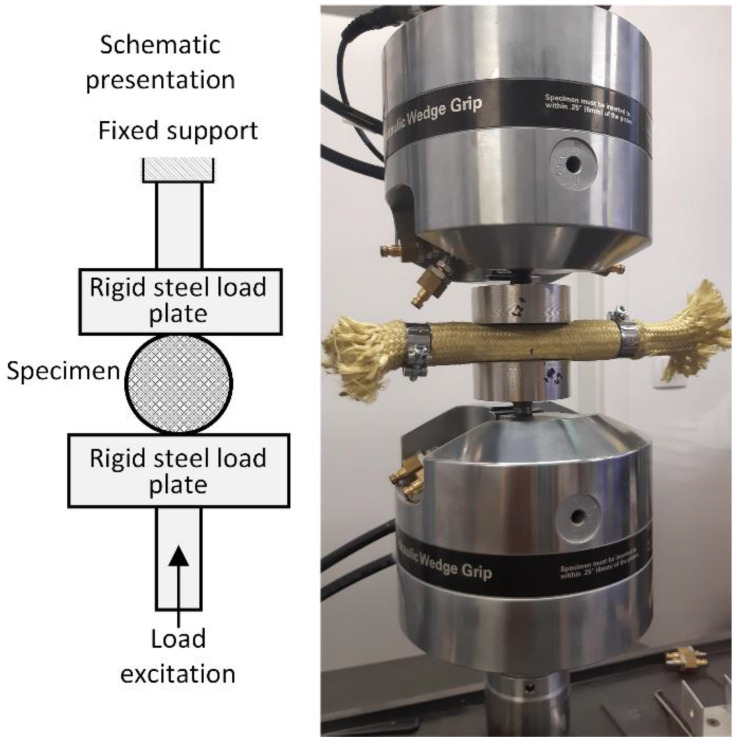
Application of compressive load to the sleeve in radial direction.

Each specimen was tested under the same loading conditions. Three arbitrarily selected excitation frequencies across several decades were used to test damping performance of the materials under investigation. The excitation frequencies were 0.08 Hz, 0.8 Hz, and 8 Hz. Compressive load was applied directly to the sleeve in radial direction, as shown schematically in Figure 9.

#### 2.3.5. Base Material

**Figure 10:**
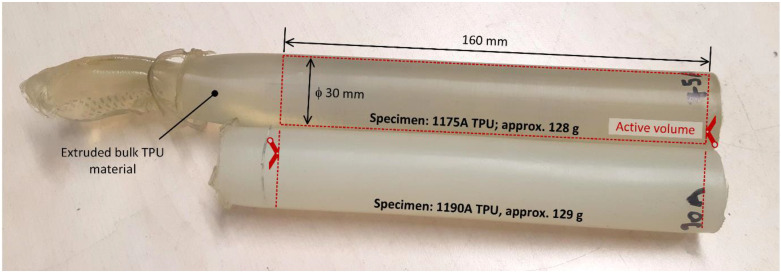
Experimental investigation of properties of base material—bulk material specimens.

The properties of the base material were determined by testing bulk TPU specimens. Raw granular material was used to prepare specimens by extrusion manufacturing process to the corresponding diameter and cut to final length, as shown in Figure 10. In order to compare the results and to evaluate possible effects of the sleeves on the damping performance of the base material, both bulk specimens with and without aramid braided fiber sleeves were made. Bulk material that was put into the sleeve was secured by two stainless steel clamps and two plugs, as shown for the granular material in Figure 6. Afterward, each bulk material specimen was placed between the two load plates and prestressed to 50 N. This is necessary to monitor the force during unloading, which in turn allows the recording of the delay between the excitation and the response of the material. The load was applied as a continuous cycle with a maximum relative displacement (strain) of 10% from the initial point set after preloading. The procedure was repeated for each of the three frequencies and for all specimens.

#### 2.3.6. Granular Material

The compression test on the 100 kN load frame was used to characterize the material’s damping behavior of the filled braided synthetic fiber sleeve specimens. Each specimen was positioned between the two load plates and prestressed with an initial force of 150 N. Again, the preload is needed to monitor the force during unloading and to record the delay between excitation and material response. Specimens were loaded with the maximum displacement, set at 10% of the initial point established by preloading. The excitation frequencies were 0.08 Hz, 0.8 Hz, and 8 Hz.

## 3. Results

We developed a new energy parameter to quantitatively evaluate and later compare the ability of each specimen category and the base material to dissipate energy.

Vibration damping generally refers to the loss of energy due to friction (i.e., internal friction including various microscopic processes such as dislocation motion and polymer chain motion) and mechanical energy (i.e., inelastic or viscoelastic deformation) that, in time, reduce the intensity of vibration [17]. Damping performance can be expressed by several variables, such as specific damping capacity, Q-factor, damping ratio, and loss factor [51]. The latter is one of the most commonly used, and it is defined as the specific damping capacity per radian of the damping cycle. The specific damping capacity is obtained by calculating the shape and area of the measured stress–strain (or force–displacement) hysteresis loop [13] as given by Equation (10).

(10) $$\eta =\frac{\Delta U}{U_{\max}}$$

where ΔU represents the energy loss per cycle and $U_{\max}$ represents the total available (reversible) energy of the system. The loss factor can equivalently be defined as the phase angle between strain and stress or as the ratio of loss and storage moduli, as further explained in the paper by Sarlin [13]. Analyses to determine the loss factor can be performed in either the time or the frequency domain: here, it is performed in the time domain.

**Figure 11:**
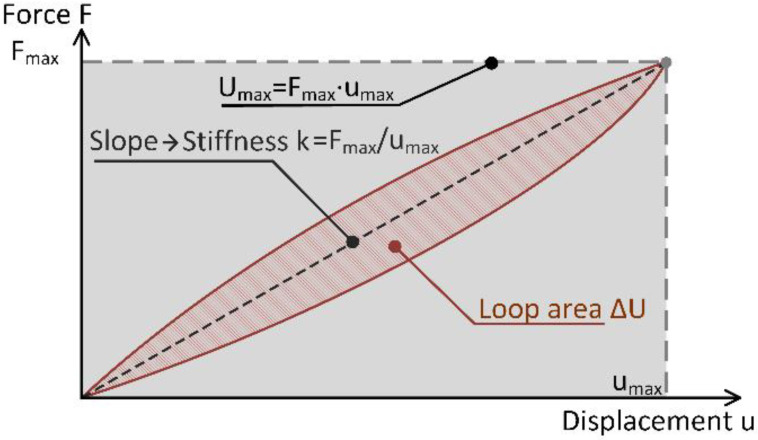
Graphical representation of relation between the $U_{\max}$ and the stiffness k.

**Figure A1:**
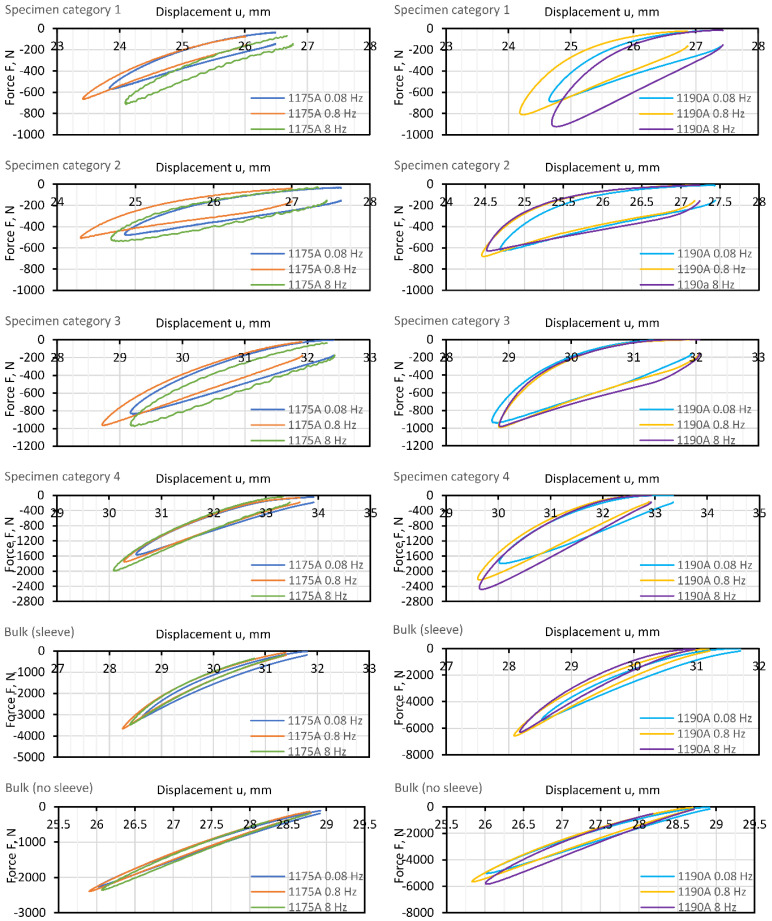
Force–displacement curves after the first load cycle as recorded for all Specimen categories at all three frequencies.

As follows from Equation (10), good vibration attenuation is achieved when the ratio is high. However, this does not guarantee that the material or the included damping technology can also deliver other design features, such as target stiffness and minimum weight. The last two are important constraints in the design of lightweight structures; thus, it is assumed that they need to be considered together with the damping performance parameter. Hence, the ratio from Equation (10) has been modified as follows from Equation (11).

(11) $$\eta^{*}=\frac{\Delta U}{U_{max}}\frac{1}{m}$$

where $\eta^{*}$ now represents lightweight—design-oriented specific damping capacity, with m representing the mass of the contributing damping material that should remain low. Normally, stiffness denotes resistance to elastic deformation. Based on the observed material response the $U_{\max}$ from Equation (11) is essentially an indirect measure of the stiffness, as it is calculated based on the maximum force (stress) measured at the maximum deflection (strain) of material, as depicted in Figure 11. Actual values, as recorded during the tests on specimens, can be found in Appendix A (Figure A1).

With the stiffness being intrinsic to the $U_{\max}$, it is assumed that Equation (11) can be further developed, and the final form of the design-oriented specific damping capacity parameter $\eta_{D}$, as follows from Equation (12), can be introduced.

(12) $$\eta_{D}=\frac{\Delta U}{k}\frac{1}{m}$$

It should be noted that Equation (12) provides a better design-oriented interpretation of damping performance compared to Equation (11). Here, however, it should be pointed out that the maximal measured displacement, $u_{\max}^{2}$, included in the relation $U_{\max}=k\cdot u_{\max}^{2}$, has been conveniently omitted from Equation (11). Hence, the first part of the Equation (12) represents the ratio of the energy dissipation and the stiffness, and the last part denotes the assumed design-specific content. The results presented in the following section will prove that the proposed design parameter $\eta_{D}$ offers meaningful information on damping and design performance, namely high values for better energy dissipation requiring less material (weight) and low values for the poor ratio between the damping performance and gained weight.

### Comparison of Design Parameter for Different Samples

**Figure 12:**
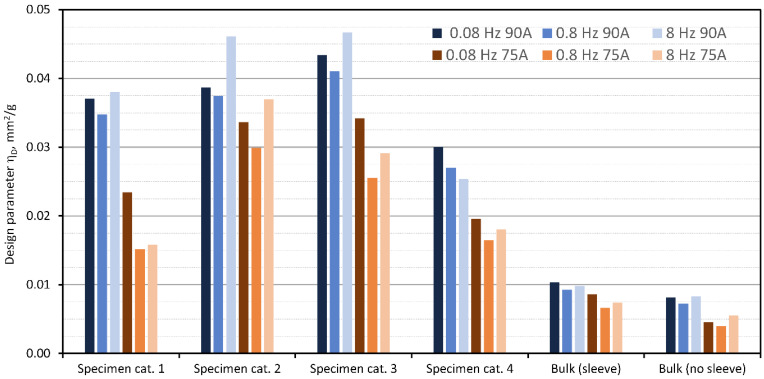
Calculated design parameter $\eta_{D}$ for each testing condition and both materials.

Following Equation (12), the collected measurements were used to calculate the values of the design parameter for all tested specimens. Results are graphically presented in Figure 12, where for better interpretation, both materials at all tested excitation frequencies are combined into one chart.

The results presented in Figure 12 show that the maximum value of 0.0467 mm^2^/g of the design parameter was observed with the specimen in the Specimen category 3 at 8 Hz and a Shore hardness of 90A of the base material. In contrast, the lowest value of 0.0040 mm^2^/g of the design parameter was observed for the bulk material specimen without a sleeve at 0.8 Hz and a Shore hardness of 75A. It should be noted that the second highest value (0.0461 mm^2^/g) is only 1% lower than the highest value, and it was observed with the specimen in the Specimen category 2 at 8 Hz and a Shore hardness of 90A of the base material. Noticeably, the bulk specimens performed the worst. Between the two bulk specimen setups (with and without aramid sleeve), bulk specimens with sleeve performed up to 50% better as compared to specimens without the sleeve (global minimum at 0.004 mm^2^/g). This is because the sleeve supports and confines the bulk material, which in turn allows a considerable increase in hydrostatic pressure as compared to the bulk without a sleeve, and this is important as it affects the damping performance.

Comparing Specimen category 1 and Specimen category 2, it is evident that lubrication improved damping performance. It is assumed that this is due to the better flowability of the granular material, which allows the formation of a larger force network and easier rearrangement of the force network during dynamic loading conditions.

**Figure 13:**
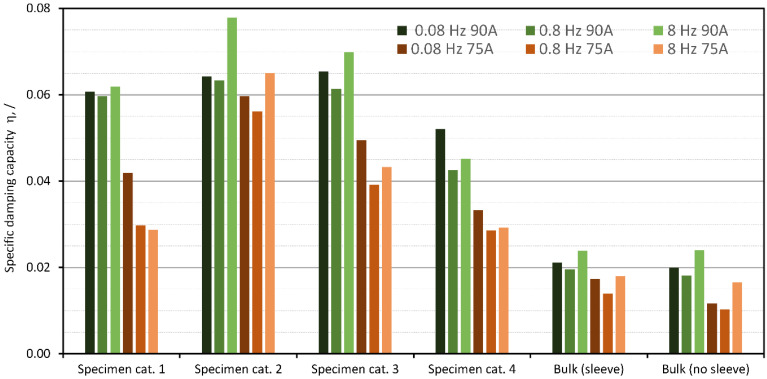
Damping performance in terms of energy dissipation presented by specific damping capacity parameter η.

It can be further investigated how the considered stiffness and mass affect the results and the estimation of the damping performance. To test this assumption, values of the specific damping capacity η from Equation (10) were calculated. The results are shown in Figure 13. In this way, damping performance is considered merely as a relationship between the dissipated energy and the total available energy of the system without considering the effect of mass.

Figure 13 confirms that the test specimens with specific initial prestress perform better compared to the other four categories of test specimens, especially at 8 Hz. However, comparing Figure 12 and Figure 13 quantitatively, the effect of the design qualifiers, namely the mass and the stiffness, can be noted. Figure 13 shows that the lubricated specimens of any hardness without prestress provided the best damping performance, but as shown in Figure 12, the lubricated and the prestressed specimen (e.g., Specimen category 3, material 1190A) offered better performance when mass and stiffness were considered. In addition, the bulk specimens performed better relative to the other specimens when mass and stiffness were not considered. It can be concluded that the introduced design parameter can be used to evaluate design-oriented damping performance because it does not favor any particular damping technology. However, it can be used to optimize the performance of vibration-damping elements that use the same damping technology as the Dissipative bulk and granular system technology presented here.

**Figure 14:**
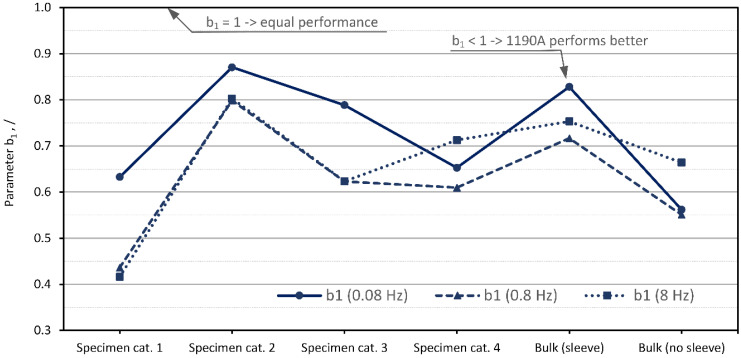
Damping performance with respect to the Shore hardness of the base material (75A vs. 90A).

Next, the effect of the base material on vibration damping is examined. This is important for a better understanding of the granular technology and to further fine-tune the available damping performance. Following the study of Bek [27], two hardness levels of the same TPU material were used for this investigation. Bek showed that within the limits of the study, a material with a Shore hardness of 90A performed better compared to 75A due to the higher impact of the hydrostatic pressure on the internal damping performance. However, it should be noted that Bek used a different experimental setup and had different research objectives. In this paper, the effect of base material on vibration-damping performance was evaluated by following the value of the introduced material parameter $b_{1}$, which is calculated for the two tested materials. Parameter $b_{1}$ is defined by the ratio of the design parameters as follows from Equation (13).

(13) $$b_{1i,j}=\frac{\eta_{D1_{75Ai},j}}{\eta_{D1_{90Ai},j}}\;\{\begin{array}{cc} for & \begin{array}{c} i=Specimen\;cat.1,Specimen\;cat.2,Specimen\;cat.3,Specimen\;cat.4\;\\ j=0.08\;\mathrm{Hz},\;0.8\;\mathrm{Hz},\;8\;\mathrm{Hz} \end{array} \end{array}$$

where $b_{1i,j}$ represent values of the parameter for all specimens and tested frequencies. Higher values (i.e., around value 1.0) indicate similar performance, whereas lower values of $b_{1}$ indicate better performance of the harder 90A granular material. Values of parameter $b_{1}$ are graphically presented in Figure 14.

The results in Figure 14 show that, with the exception of Specimen category 2, the base material with a Shore hardness of 90A performed better than the material with a Shore hardness of 75A. The result of the bulk material without the sleeve showed negligible differences between material performance—both hardness and frequency wise.

## 4. Discussion

The simplest option to control the vibration-damping performance of materials is by means of materials science. In general, there are a variety of possibilities in which polymers can be blended together in order to achieve the most satisfactory properties, such as intrinsic natural frequency, vibration damping, and stiffness, to meet the requirements of a particular application. However, since the technical part of the blending process is usually demanding, requires excellent knowledge of chemistry and chemical processes, and consumes expensive raw materials and energy, manufacturers often rely only on a few selected and proven compounds and technologies.

In this paper, an alternative to the blending process is presented, using granular material instead of bulk material. The results presented in the previous sections show that such technology can provide up to four times better vibration-damping performance as compared to the same type of material in bulk form. The results are encouraging, especially considering the amount of granular material used.

To address the main research premise, namely, to confirm experimentally that the interactions between the molecules at the molecular scale and the interactions between granules in a granular system at the macro scale complement each other, we identified and qualitatively evaluated (see Table 3) three main mechanisms affecting the damping performance of GDE elements. The three damping mechanisms are as follows:

- Material internal damping (molecular scale);
- Influence of hydrostatic pressure (molecular-macro scale);
- Force chain network (macro scale).

**Table 3:** Qualitative evaluation of effects of the damping mechanism on the GDE damping performance.

	Force Chain	Internal Damping	Hydrostatic Pressure
Specimen cat. 1	●●●○	●○○○	●○○○
Specimen cat. 2	●●●●	●○○○	●○○○
Specimen cat. 3	●●●●	●○○○	●●○○
Specimen cat. 4	●●●○	●○○○	●●○○
Bulk (sleeve)	○○○○	●●●○	●●●○
Bulk (no sleeve)	○○○○	●●●●	○○○○

The experimental results show that damping mechanisms are expressed differently in individual specimen categories. Specimen category 1 and Specimen category 2 have no prestress; therefore, effects of hydrostatic pressure and internal damping contribute considerably less to the overall performance as compared to the recognized main, macro scale, and force-chain network mechanism. By increasing the level of prestress, e.g., Specimen category 3 and 4, the effect of hydrostatic pressure becomes more apparent, and the effect of the force-chain network is decreased. The damping performance of the unconfined bulk material can only be affected at the molecular scale by the internal damping mechanism, whereas the damping performance of the constrained bulk material can be affected by both the internal damping mechanisms and the effects of hydrostatic pressure. The force-chain network can only appear in granular materials; therefore, bulk material performance remains unaffected by this mechanism.

Furthermore, the results for the granular material show that the three damping mechanisms complement each other, but here, due to the relatively low level of applied hydrostatic pressure, the force-chain network mechanism predominates. After cross-analyzing the experimental results, several conclusions supporting the above qualitative evaluation can be drawn. The findings can be divided into five categories as follows.

**Lubrication** of granular material is important for several reasons. Administrating a small amount of petroleum jelly to the granular material improved the specimen preparation process due to better flowability. Namely, the petroleum jelly importantly reduced the force required to fill the aramid sleeve by reducing the friction between the granules. Consequently, a better arrangement of the granules was achieved during preparation than in the non-lubricated specimens. The latter is confirmed by comparing the damping performance results of lubricated and non-lubricated specimens, where the latter performed better than the former. The difference in the damping performance indicates that the non-lubricated granular structure was not sufficiently restructured so the formation of the force-chain network was significantly reduced.

**Initial prestress** plays an important role in establishing and maintaining the force-chain network. The results show that granular specimens in Specimen category 3 with an initial prestress corresponding to 80% of the volume ratio performed better than specimens in Specimen category 2 without a prestress, corresponding to a volume/void ratio of about 65%. On the other hand, a much higher level of prestress, as used with Specimen category 4 (corresponding to 93% of the volume ratio), can negatively affect performance. This is because, here, the hydrostatic pressure mechanism prevails over the force-chain network damping mechanism. Moreover, high pressure drastically reduces the possibility of reformation of the force-chain network during the dynamic loading, due to which the share of the force-chain network damping mechanism is drastically decreased.

**Base material** of granulate. This study confirms the observations of Bek [27] that the 1190A TPU material is more sensitive to pressure and generally attenuates external excitations better than the 1175AW TPU material. Since the Shore A value essentially represents the elastic modulus of the tested material, it can be concluded that the 1190A TPU material is stiffer and harder than the 1175AW TPU material. The results show that for the same preload, the harder material performs better, which is overall and relative to the preload. This effect is believed to be due to both: formation of stronger force-chain pairs—the development of a larger and more stable force-chain network throughout the volume (flowability of the 1190A) and higher internal damping (different time and frequency-dependent properties [27]). Effects related to base material were further enhanced by the addition of lubricant, which can be observed by comparing the performance of the unlubricated and the lubricated specimens. More specifically, the ratio between the design parameter of the 1190A and the 1175A material decreased in favor of the 1175A material when lubricant was added to the granular material. It should be noted that, for objective reasons, the contribution of each mechanism to the overall performance can only be qualitatively estimated. It can be concluded that the optimum damping performance can be achieved by choosing the right combination of material, initial prestress, and lubricant.

**Confined volume** is needed to hold the granular material in place. For this purpose, high-strength aramid braided sleeves were used. This technology allowed both the application of prestress and the application of an external radial force acting directly on the granular material, which would be impossible with rigid containers. Bulk material corresponding to 100% of the volume ratio was also filled into a sleeve and tested under the same loading conditions. Bulk material without a sleeve was tested as a reference. The results show that the performance in the latter two cases was significantly lower than the performance of the granular specimens. This is because neither of the bulk specimens take advantage of the force-chain network damping mechanism. When comparing the two bulk specimen categories, it is obvious that adding the sleeve additionally introduced a damping mechanism, namely a mechanism related to hydrostatic pressure. This is why the performance of the enclosed bulk specimen was slightly better compared to the sleeveless bulk specimen.

This study shows that the patented Dissipative Bulk and Granular System, originally developed for extreme prestresses, can improve damping performance even at much lower prestresses. This opens up a wide range of potential practical applications in the automotive, transportation, and energy industries, to name a few. To this end, further studies are needed to gain a deeper understanding of this promising vibration-damping technology.

## Figures and Tables

**Table 1:** A list of selected physical properties provided by the Basf^®^ manufacturer for the 1190A and 1175AW TPU Elastollan^®^ measured on injection-molded samples.

Physical Property	ASTM Test Method	Units	Typical Value for 1190A	Typical Value for 1175A
Relative density	ASTM D 792	g/cm^3^	1.14	1.13
Shore Hardness	ASTM D 2240	Shore A	90A	75A
E-Modulus	ASTM D 412	MPa	31 (4500 psi)	9.0 (1300 psi)
Flexural Modulus	ASTM D 790	MPa	29 (4200 psi)	10.3 (1500 psi)
Tear Strength	ASTM D 624, Die C	kg/cm	130 (730 lb/in)	82.0 (460 lb/in)

**Table 2:** Details on the employed volume ratios and used granular material.

Specimen Category	Material	Condition	Intial Pre-Stress	κ [%]	Equivalent Mass of Granular Material [g]
1	1175A	Dry	*No*	62.1	74.40
2	1175A	Lubricated	*No*	66.7	79.91
3	1175A	Lubricated	*Yes*	80.0	95.75
4	1175A	Lubricated	*Yes*	93.0	119.81
1	1190A	Dry	*No*	62.7	75.79
2	1190A	Lubricated	*No*	64.3	77.72
3	1190A	Lubricated	*Yes*	80.0	96.70
4	1190A	Lubricated	*Yes*	93.0	120.87

## Data Availability

Not applicable.
